# Supplementary material for: Regulatory T Cells Exhibit Interleukin-33-Dependent Migratory Behavior during Skin Barrier Disruption
Source: Int J Mol Sci. 2021 Jul 12;22(14):7443. doi: 10.3390/ijms22147443 (PMC8304226; doi:10.3390/ijms22147443)
Supplement: Supplementary file 1 [file ijms-22-07443-s001.zip › ijms-1273555-supplementary.pdf]

Regulatory T Cells Exhibit Interleukin-33-Dependent Migratory Behavior during Skin Barrier Disruption

Sumika Toyama [1], Catharina Sagita Moniaga [1], Susumu Nakae [2], Masaru Kurosawa [1], Hideoki Ogawa [1],
Mitsutoshi Tominaga [1] and Kenji Takamori [1,3,*]

[1] Juntendo Itch Research Center (JIRC), Institute for Environmental and Gender Specific Medicine,
Juntendo University Graduate School of Medicine, 2-1-1 Tomioka, Urayasu, Chiba 279-0021, Japan;
su-toyama@juntendo.ac.jp (S.T.); m-catharina@juntendo.ac.jp (C.S.M.); mskurosa@juntendo.ac.jp (M.K.); ogawa@juntendo.ac.jp (H.O.); tominaga@juntendo.ac.jp (M.T.)
[2] Graduate School of Integrated Sciences for Life, Hiroshima University, 1-4-4 Kagamiyama,
Higashi-Hiroshima, Hiroshima 739-8528, Japan; snakae@hiroshima-u.ac.jp
[3] Department of Dermatology, Juntendo University Urayasu Hospital, 2-1-1 Tomioka, Urayasu,
Chiba 279-0021, Japan
* Correspondence: ktakamor@juntendo.ac.jp; Tel.: +81-47-353-3171; Fax: +81-47-353-3178

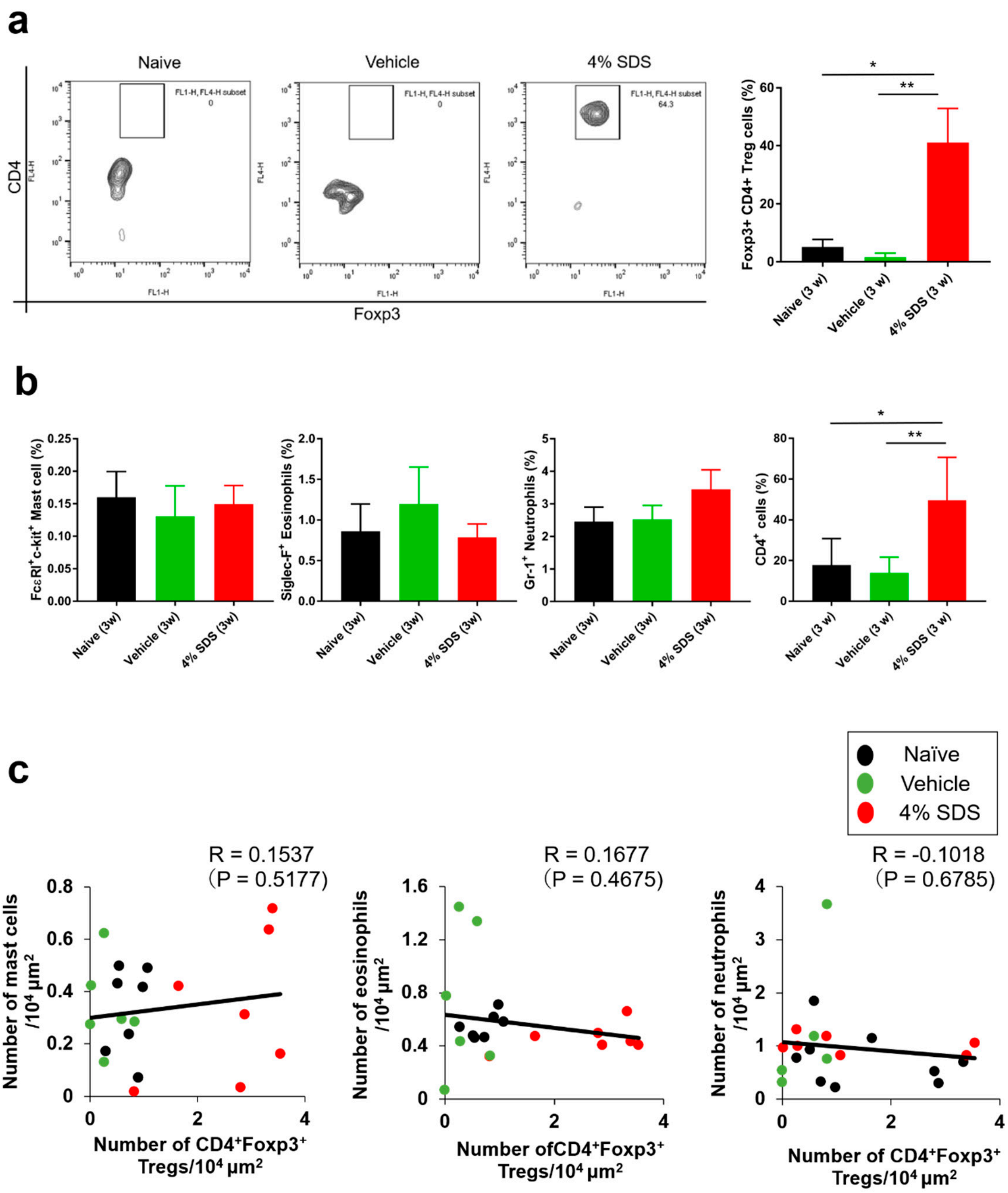

Supplementary Figure S1. Correlation analyses between Treg infiltration and mast cell, eosinophil and neutrophil infiltration. a; The proportion of $CD4^{+}Foxp3^{+}$ Treg cells among $CD25^{+}$ cells was assessed by flow cytometry. Data show representative results from 8 mice of naïve, vehicle-treated and 4% SDS-treated skins per experiment and from

at least two independent experiments, each of which gave similar results. b; Percentage of FcεRI^{+}c-kit^{+} mast cells, Siglec-F^{+} eosinophils, Gr-1^{+} Neutrophils and CD4^{+} cells in the skin. Data show representative results from 8-12 mice of naïve, vehicle-treated and 4% SDS-treated skins per experiment and from at least two independent experiments, each of which gave similar results. *P<0.05 and ** P<0.01 compared with naïve or vehicle-treated mice by the Kruskal-Wallis with Dunn's multiple-comparison test. c; Scatterplots of correlations between Treg number and the number of mast cells, eosinophils or neutrophils; N=21 (Naïve; N=7, Vehicle; N=6, 4% SDS; N=8). Coefficients (r) were determined by Spearman rank correlation tests. P<0.05 indicated significance. Results are shown as the mean ± SEM of two independent experiments.

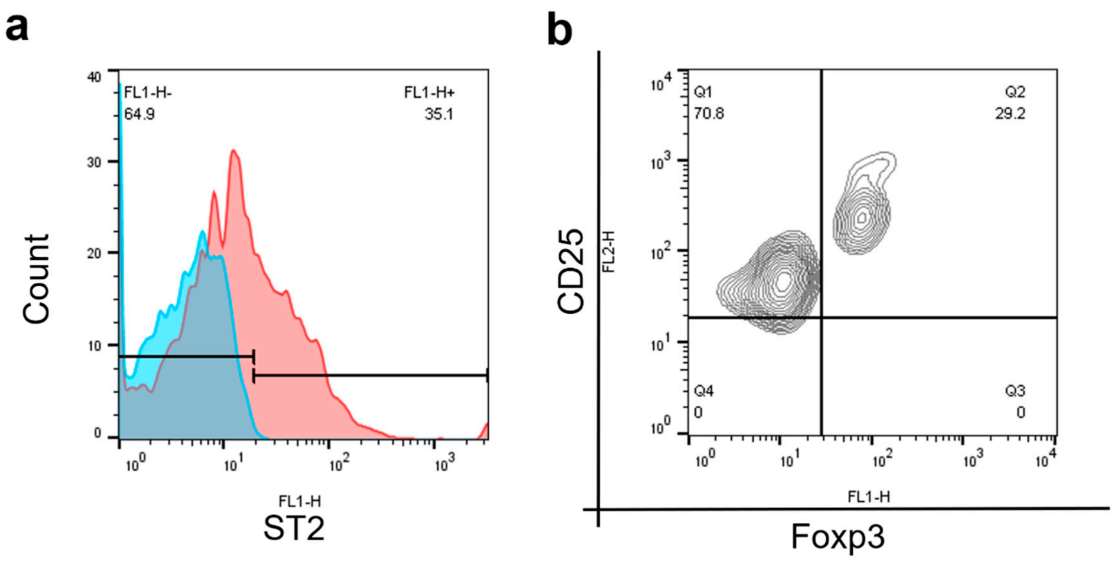

Supplementary Figure S2. Expression analysis of ST2 or Foxp3 in isolated CD4$^+$CD25$^+$ Tregs. a; The proportion of ST2$^+$ Treg cells among CD4$^+$ CD25$^+$ T cells was assessed by flow cytometry. Data show representative results from 4 mice of naïve mice in spleen per experiment and from at least two independent experiments, each of which gave similar results. b; The proportion of Foxp3$^+$ Treg cells among CD4$^+$ CD25$^+$ T cells was assessed by flow cytometry. Data show representative results from 4 mice of naïve mice in spleen per experiment and from at least two independent experiments, each of which gave similar results.

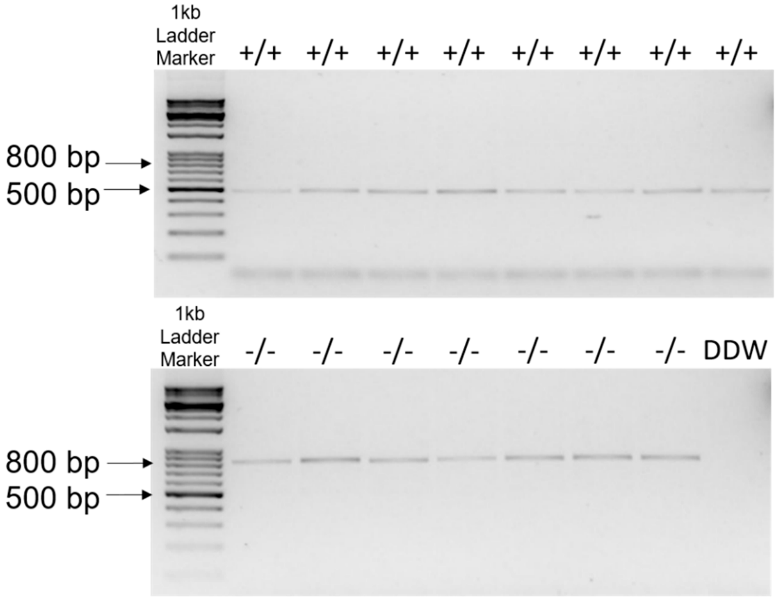

Supplementary Figure S3. Genotyping of IL-33$^{-/-}$ mice. PCR genotyping of IL-33$^{-/-}$ mouse tail DNA. Wild type; 489 bp, Knockout; 850 bp.
